# Seasonality Role on the Phenolics from Cultivated *Baccharis dracunculifolia*

**DOI:** 10.1093/ecam/nep077

**Published:** 2011-06-23

**Authors:** João Paulo B. de Sousa, Mateus F. Leite, Renata F. Jorge, Dimas O. Resende, Ademar A. da Silva Filho, Niege A. J. C. Furtado, Ademilson E. E. Soares, Augusto C. C. Spadaro, Pedro Melillo de Magalhães, Jairo K. Bastos

**Affiliations:** ^1^Faculdade de Ciências Farmacêuticas de Ribeirão Preto, Universidade de São Paulo, Avenida do Café, s/n, 14040-903, Ribeirão Preto, Brazil; ^2^Núcleo de Pesquisa em Ciências Exatas e Tecnológicas, Universidade de Franca, Avenida Dr. Armando Salles Oliveira, 201, 14404-600, Brazil; ^3^Departamento de Genética, Faculdade de Medicina de Ribeirão Preto, Universidade de São Paulo, Avenida do Café, s/n, 14049-900, Ribeirão Preto, SP, Brazil; ^4^Centro Pluridisciplinar de Pesquisas Químicas, Biológicas e Agrícolas – UNICAMP, 13081-970, Campinas, Brazil

## Abstract

*Baccharis dracunculifolia* is the source of Brazilian green propolis (BGP). Considering the broad spectrum of biological activities attributed to green proplis, *B. dracunculifolia* has a great potential for the development of new cosmetic and pharmaceutical products. In this work, the cultivation of 10 different populations of native *B. dracunculifolia* had been undertaken aiming to determine the role of seasonality on its phenolic compounds. For this purpose, fruits of this plant were collected from populations of 10 different regions, and 100 individuals of each population were cultivated in an experimental area of 1800 m^2^. With respect to cultivation, the yields of dry plant, essential oil and crude extract were measured monthly resulting in mean values of 399 ± 80 g, 0.6 ± 0.1% and 20 ± 4%, respectively. The HPLC analysis allowed detecting seven phenolic compounds: caffeic acid, ferulic acid, aromadendrin-4′-methyl ether (AME), isosakuranetin, artepillin C, baccharin and 2-dimethyl-6-carboxyethenyl-2H-1-benzopyran acid, which were the major ones throughout the 1-year monthly analysis. Caffeic acid was detected in all cultivated populations with mean of 4.0%. AME displayed the wide variation in relation to other compounds showing means values of 0.65 ± 0.13% at last quarter. Isosakuranetin and artepillin C showed increasing concentrations with values between 0% and 1.4% and 0% and 1.09%, respectively. The obtained results allow suggesting that the best time for harvesting this plant, in order to obtain good qualitative and quantitative results for these phenolic compounds, is between December and April.

## 1. Introduction

*Baccharis dracunculifolia* D. C. (Asteraceae) is a native plant from Brazil commonly known as “Alecrim do campo” and “Vassoura”. This plant is well known for its interaction with insects, mainly *Apis mellifera* L., and for bearing a wide range of secondary metabolites. Its leaves are punctuated with secretory thricomes that are rich in secondary metabolites, as well as secretory ducts that produce and store essential oils and phenolic compounds. *Baccharis dracunculifolia* secondary metabolites are collected by *A. mellifera* to produce Brazilian green propolis (BGP) [1], which is of great importance for food and pharmaceutical industries [2] as it displays anticancer [3], antibacterial [4], anti-inflammatory [5] and antiulcer [6] properties among others. Lemos et al. [7] described the gastric protective effect of the hydroalcoholic extract of *B. dracunculifolia* aerial parts. Fukuda et al. [8] reported the cytotoxic activity of *B. dracunculifolia* constituents. Da Silva Filho et al. [9] showed the presence of flavonoids [isosakuranetin, aromadendrin-4′-methyl ether (AME)] and cinnamic acid derivatives (caffeic acid, *p*-coumaric acid, ferulic acid) with trypanocidal activity. Munari et al. [10] reported the antimutagenic activity of the hydroalcoholic extract of the leaves of this plant. Akao et al. [11] showed that prenylated *p*-coumaric acid derivatives (artepillin C, drupanin and baccharin) exhibited antitumor properties. Missima et al. [12] identified diterpenes and triterpenes with immunomodulatory activity. Leitão et al. [13] reported that *B. dracunculifolia* displays anticariogenic activity. Klopell et al. [14] found that (*E*)-nerolidol, the major constituent of the volatile fraction, stood out for antiulcer activity.

It is important to point out that honey is another major bee product, and recently there were three reported works: the inhibition of lipid peroxidation in biological systems [15], the antiseptic agent in wound care [16] and the enhancement of immune function and antitumor activity [17]. In addition, seasonal variation, chemical composition and antioxidant activity of Brazilian propolis samples were reported as well [18]. Forty phenolic substances were identified, in different concentrations, from Brazilian propolis extracts produced in three distinct regions. It is well known that *B. dracunculifolia* is the main botanical source of BGP. Therefore, considering that the majority of reported works with *B. dracunculifolia* were undertaken with native plants and that this plant has a great potential for the development of new products, the aim of this work was to evaluate the seasonality role in the phenols chemical profile of 10 different populations of *B. dracunculifolia* cultivated during 1 year. It would not only allow the selection of the *B. dracunculifolia* population bearing higher production of the phenolic compounds, but also to determine the best timing of plant harvesting.

## 2. Methods

### 2.1. Reagents and Solvents

Organic solvents for HPLC analyses were purchased from Mallinckrodt Co. (Xalostoc, Mexico) and filtered through 0.45-*μ*m cellulose membranes prior to use. Water was purified using Milli-Q-plus filter systems (Millipore, Bedford, MA, USA). Caffeic acid and ferulic acid were bought from Acros Organics (Morris Plains, NJ, USA). The internal standard (IS), 3,4-dimethoxybenzaldehyde (veratraldehyde) was purchased from Merck (Darmstadt, Germany). The phenolic AME, isosakuranetin, artepillin C, baccharin and 2,2-dimethyl-6-carboxyethenyl-2H-1-benzopyran acid (DCBEN) were isolated and identified in our laboratory from either *B. dracunculifolia* or propolis samples [19, 20]. The purity of each standard was determined by both HPLC and ^13^C NMR to be higher than 96%.

### 2.2. Cultivation and Sampling

Initially, the fruits of *B. dracunculifolia* were collected from populations of 10 different regions of Brazil in their natural habitat (Table 1). Professor Nelson Ivo Matzenbacher authenticated the plant material. The fruits were first germinated in a nursery and then propagated under glasshouse conditions for 30 days. The obtained seedlings were transplanted to the experimental field area of Chemical, Biological and Agricultural Pluridisciplinary Research Center (CPQBA), University of Campinas, São Paulo, in January 2004. The field was divided into four replications. Each replication was composed of 10 blocks. Each block was composed of 25 plants, totalling 1000 cultivated plants in the area. Voucher specimens of each replication were deposited in the herbarium at CPQBA-UNICAMP (No. 1298). The cultivation experiment was carried out in an experimental area of 1800 m^2^ (22^˚^48′S, 47^˚^03′W, altitude 669 m), at the University of Campinas—CPQBA, Brazil. *Baccharis dracunculifolia* was cultivated according to the Good Agriculture Practices (GAPs) and the cultivated area was kept free of weeds by manual procedures and no herbicides were used.

The sampling of the leaves started 4 months later and it was undertaken between May 2004 and April 2005. Ten randomized samples consisting of branches measuring 20 cm each, containing both buds (30%) and adult leaves (70%) from individual plants, representing each replication, were collected as homogeneous samples from each population. The homogenous samples were harvested monthly to determine both chemical profile and seasonal role on the major phenolic constituents.

### 2.3. Sample Preparation and Chromatographic Analysis

The sample preparation was undertaken following the analytical method previously developed [21]. The buds and adult leaves from 10 dried collected branches of *B. dracunculifolia* were removed and powdered using a knife mill. To a homogeneous sample of 500 mg, 20 ml of 90% ethanol containing 300 *μ*g/ml of the internal standard in 125 ml Erlenmeyer flasks was added. The solution was stirred at 170 r.p.m. and 40^˚^C on a shaker (Innova 4300, New Brunswick Scientific, Edison, NJ, USA). After 2 h of extraction, the flasks were cooled to room temperature and filtered through analytical filter papers. A 1.0 ml aliquot of the extracts was then filtered through a Millex-LCR-PTFE (Millipore, Bedford, MA, USA *0.45* *μ*m × 13 mm i.d.) and transferred to an appropriate vial for automatic injection, and a 15-*μ*l aliquot was injected into the HPLC system, which was described by Sousa et al. [21].

Veratraldehyde was used as internal standard, and it was added to the extracting solvent prior to extraction. The spectral data from the photodiode array detector were collected within 60 min over the 265–320 nm range of the absorption spectrum, and the chromatograms were plotted at 280 nm. Peaks were assigned according to their retention times and by co-elution with authentic standards, as well as based on UV spectra for both the standards and samples under the same chromatographic conditions.

### 2.4. Quantitative Analysis and Statistical Studies

The calibration curves were prepared in the concentration range expected of each compound in *B. dracunculifolia* sample, ranging from 25 to 1200 *μ*g/ml. The linearity was investigated by calculation of the regression plots by the least squares and it was expressed by the determination coefficient (*R* ^2^) showing values ranging from 0.9982 to 0.9998. Absolute concentrations of four compounds (acid caffeic, AME, isosakuranetin and artepillin C) in the *B. dracunculifolia* samples were calculated based on the phenolic area/IS area. Regarding the chromatographic profile of the hydroalcoholic extracts, the relative percentages of each peak of interest were obtained monthly, taking into account the area percentage.

After checking for normality (Kolmogorov-Smirnov test) and homogeneity of the variances (Bartlett's test), the inter-group variation of different parameter was estimated by the analysis of variance (ANOVA). These ANOVA analyses were then completed by Tukey's multiple range tests, in order to locate the differences [22]. Thus, qualitative treatments were compared by Tukey test, with a probability of 95% and significance levels of 5% (*P* < .05) for comparative studies among populations. It was also considered the probability of 99% and significance levels of 1% (*P* < .01) for comparison among months and considering each population as well. Statistics calculations, as well as graphic representation were prepared by using GraphPad Prim^®^ (v. 4.0) and additional calculations were carried out with aid of Microsoft^®^ Excel 2003.

## 3. Results

### 3.1. Agronomy Aspects

*Baccharis dracunculifolia* was cultivated rapidly and developed from the production of seedlings in nursery, showing excessive and intense growth in an interval of 2 months. At the fourth month it was about 0.8 m in height, achieving 2.5–3.0 m in 1 year, despite of no use of chemical fertilizers during the plant development. The productive potential of the species expressed as amount of dry biomass per plant was variable depending on the population. According to the obtained results, the mean of yielding of plant dry biomass, after 16 months of seeding and considering 10 populations, was 399 ± 80 g. The Paraguaçu-MG (302 ± 34 g) and Colombo 1-PR (584 ± 75 g) populations displayed the lowest and the highest yield of dry biomass, respectively. Additionally, the essential oil of the dry leaves of each cultivated population was studied as well [23].

### 3.2. Chemical Composition

**Figure 1:**
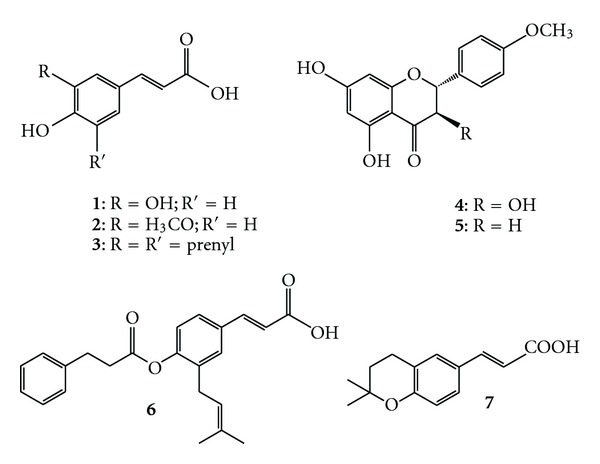
Chemical structures of main phenolics identified in extracts from the leaves of cultivated specimens of *B. dracunculifolia*. **1**: caffeic acid; **2**: ferulic acid; **3**: artepillin C; **4**: AME; **5**: isosakuranetin; **6**: baccharin and **7**: DCBEN.

The HPLC method used allowed the analysis of seven major phenolic compounds along 1 year in *B. dracunculifolia* samples: caffeic acid, ferulic acid, AME, isosakuranetin, artepillin C, baccharin and DCBEN. Chemical structures for these seven phenolics are displayed in Figure 1.

### 3.3. Seasonality and Phenolic Contents

The quantification of caffeic acid, AME, isosakuranetin and artepillin C were undertaken for all cultivated plants from 10 distinct regions. Tables 2–5 display the variation on the concentration of these four phenolic compounds between May 2004 and April 2005. Considering the mean values calculated monthly for *B. dracunculifolia* from these regions, the relative percentages of these phenolics were obtained by taking their percentages in the ethanolic extracts. The sum of these four compounds correspond to *∼*30% of the total extract, caffeic acid (23.7 ± 2.4%) being the major one, followed by AME (2.9 ± 1.1%), isosakuranetin (2.1 ± 1.2%) and artepillin C (1.3 ± 0.8%), respectively.

Caffeic acid and AME were detected in all the studied populations during the entire year (Tables 2 and 3). Artepillin C was found in most of the studied populations, with exception of the one from Colombo 1 (Table 5). Isosakuranetin was the phenolic that displayed larger qualitative variation, since it was detected mainly in the last 6 months, between November and April of 2005 (Table 4). Ferulic acid, baccharin and DCBEN, were found in almost every period of analysis, but in concentrations lower than the limit of quantification previously established [21].

**Figure 2:**
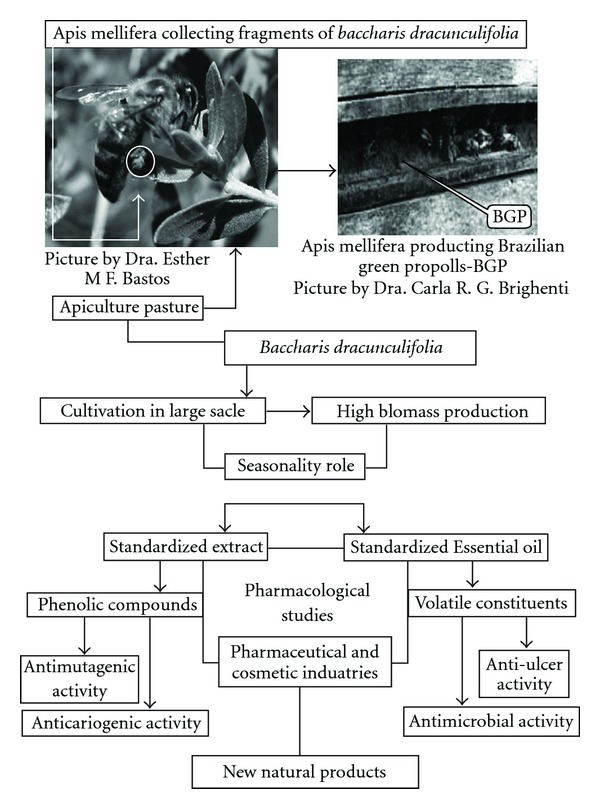
Diagram demonstrating the potential of *B. dracunculifolia* for both pharmaceutical and cosmetic industries in the production of BGP, standardized extracts and essential oil.

Taking into account the agronomic aspect, chemical composition, seasonality role and phenolic contents, the results of this work are summarized in Figure 2 showing the potential of *B. dracunculifolia* for both pharmaceutical and cosmetic industries in the production not only of BGP, but also the standardized extracts and essential oil.

## 4. Discussion

### 4.1. Cultivation of B. dracunculifolia

Seasonality is an important factor to be considered in the cultivation of *B. dracunculifolia* because rainfall availability, humidity, temperature, nutrients as well as herbivory or attack of pathogens are basic factors that along with environment can influence biosynthesis of plant secondary metabolites. *Baccharis dracunculifolia* have drawn great attention among agronomists, chemists and pharmacists, aiming to develop the agro technological knowledge to allow both the acclimatization and the selection of a high productive population. In this regard, the knowledge about the best cultivation techniques is one of the first steps to develop commercial scale production.

The developed cultivation technique demonstrated to be feasible of cultivating 1000 plants in an area of 1800 m^2^. The yields of dry plant, essential oil and crude extract were measured monthly resulting in mean values of 399 g, 0.6 ± 0.1% and 20 ± 4%, respectively. Hence, the cultivation of *B. dracunculifolia* in large scale using an area of 10 000 m^2^, which is equivalent to 1 hectare, would allow to cultivate 5556 individuals furnishing, after 12 months of cultivation, about 2200 kg of dry plant, from which it could be obtained 13 kg of essential oil or 440 kg of crude extract. Therefore, it is viable to cultivate this plant in large scale for commercial use, since industries of medium and large productivity, specializing in plants, have the ability to grow at least 5 hectares of the plant of interest.

Considering *B. dracunculifolia* plant, this is the first time that cultivation studies, involving chemical composition analysis and seasonality role, have been reported. Thus, the cultivation of this species can provide biomass for phytochemical and pharmacological studies, as well as for the continuous supply of botanical raw material. Moreover, the selection of a good population for cultivation could enhance the production of desired compounds.

### 4.2. Standardized Extract and Phenolic Compounds

To obtain standardized extracts it is necessary to produce biomass with excellent quality. For that, the development of an analytical-validated method is mandatory to determine the concentration of each metabolite of interest in the plant biomass. Moreover, the analytical method is an important tool to study the influence of seasonality, to select a good population for cultivation, to determine the best time for harvesting, to develop the extraction and formulation process, to analyze the final products, to run the pre-clinical and clinical assays, among others.

Most of the works with *B. dracunculifolia* report its secondary metabolites as main source for the production of BGP. Because of that, there are researches reporting comparative studies about the chemical composition of this plant and its relationship with green propolis [24, 25]. So far, at least 100 substances in native *B. dracunculifolia* have been identified including: cinnamic acid derivatives, anthracene derivatives, phenolics, prenylated phenylpropanoids, sesquiterpenes, diterpenes, and triterpenes, among others, for which different biology activities have been found [9, 13, 20, 26].

Intake of the phenolic compounds present in both BGP and *B. dracunculifolia* as health promoter has been linked to reduced risk of colon cancer and gastrointestinal disorders [27, 28]. Caffeic, ferulic and *p*-coumaric acids are *trans*-cinnamic acids that occur naturally in their free forms, and as a family of mono or diesters with (–)-quinic acid, collectively known as chlorogenic acids (CGAs). CGAs are antioxidant components produced by plants in response to environmental stress conditions such as infection by microbial pathogens, mechanical wounding as well as excessive UV or visible light levels [29].

An important phenolic acid, present in both Brazilian propolis and *B. dracunculifolia*, is 3,5-diprenyl-4-hydroxycinnamic acid (DHCA), which is known as artepillin C. Studies have shown that DHCA inhibits lipid peroxidation and the development of pulmonary cancer in mice prevents colon cancer through the induction of cell-cycle arrest, and displays chemopreventive action in colon carcinogenesis, as well as anti-leukemic effect with low inhibitory effect on normal lymphocytes [30]. Isosakuranetin and AME, along with other flavonoids have been widely investigated [9, 13, 31], and their intake may have beneficial effects such as: increase vitamin absorption and action, help wound-healing processes, act as antioxidant, antimicrobial and immunomodulatory [32]. With respect to derivatives of *p*-coumaric and caffeolyquinic acids, a positive association of the biological activity against *Staphylococcus aureus*, *S. pneumoniae* and *Trypanosoma cruzi* was found [26]. DHCA and DCBEN, previously characterized in both BGP and *B. dracunculifolia*, were active against *T. cruzi* and *S. aureus* [33]. In addition, Barros et al. [28] demonstrated that caffeic, ferulic, *p*-coumaric and cinnamic acids possess gastro-protective activity. Therefore, the knowledge of the chemical variations of phenolic compounds in *B. dracunculifolia* becomes essential to provide raw materials of high quality for the development of new products. In this regard, the developed protocols allowed to undertake the analysis of 480 samples of *B. dracunculifolia* collected monthly during 1 year. Also, based on these results AME along with caffeic acid could be considered good chemical markers for the analysis of cultivated *B. dracunculifolia*, considering that both were found in all the samples throughout the studied year.

### 4.3. Seasonal Variation

The phenolic compounds can be considered as a chemical interface between *B. dracunculifolia* and surrounding environment, and their biosynthesis can be amended by environmental conditions. Thus, Weather and Climate Applied to Agricultural Research Center CEPAGRI-UNICAMP monitored rainfall availability, humidity and temperature of the cultivation site. The mean of temperature, considering the 12 months of the experiment, was 22.5^˚^C. The lowest mean temperature (19^˚^C) was detected between May and July and the highest (25^˚^C) was detected between January and March. The average rainfall for the year was 120 mm. The lowest average of rain (42 mm) occurred from May to July and the highest amount of rain (218 mm) occurred from January to March. The humidity did not vary significantly along the year, resulting in mean values of 60%. It is important to point out that the flowering period of *B. dracunculifolia* in this experiment occurred from May to July of 2004, which was the period that displayed both lower temperature and less amount of rain. During the flowering period wide variation in the concentration of phenolics was observed (Tables 2–5).

It is mandatory to know the role of seasonality on the chemical profile and the content of each compound of interest in a cultivated plant for pharmaceutical use, aiming to obtain either standardized extract or pure compounds, which can define the potential of individual components and give its potency on the synergistic effect, considering the major metabolites as a whole.

According to statistic studies (one-way ANOVA), the caffeic acid did not vary significantly during the period of study in both individuals and among all populations, with mean value of 4.0% (Table 2). The population from Colombo 1-Paraná displayed significant variations for artepillin C (*P* < .05) (Table 5) and AME (*P* < .05) (Table 3). On one hand, the concentrations of AME, comparing population from Colombo 1 with other populations were higher ranging between 0.45% and 1.11%, and on the other hand the concentrations of artepillin C were lower (0–0.16%). The isosakuranetin (Table 4) showed statistic difference for the populations from Paraguaçu-Minas Gerais and Colombo 1. The concentration levels for this flavonoid in Paraguaçu region were higher (0–1%; *P* < .05) in comparison with other populations, while for the population from Colombo 1, isosakuranetin was not detected.

The statistical analysis of the data, considering each population and the mean values, which were obtained among the population for the different months of the experiment, are shown in Tables 2–5 with *P* < .01. In general, the significant values found for each population were similar to the ones obtained by the calculation of the mean values given by each month. Thus, taking into account the monthly mean values it is possible inferring that caffeic acid content was higher in the months of October (4.96 ± 0.92%), January (4.93 ± 0.53%), March (5.17 ± 0.52%) and April (4.33 ± 0.50%). Isosakuranetin and artepillin C displayed higher concentrations in the months of November, December and from January through April. Likewise, the means concentration for isosakuranetin ranged from 0.49 ± 0.20 to 0.79 ± 0.32%, and for the prenylated *p*-coumaric acid derivate, it ranged from 0.25 ± 0.10 to 0.71 ± 0.21%. Regarding AME, its concentrations behaved differently from the other phenolics. The mean yield for this phenolic was about 0.7 ± 0.13% in May, decreasing to 0.4 ± 0.15% until August. In September there was an increase again to 0.75 ± 0.20%, which decreased to 0.34 ± 0.24% in January, and increased to 0.65 ± 0.13% in February, which was maintained until April. Statistically, AME content was higher in the months of May, September and February through April. Moreover, during this same period other compounds, such as ferulic acid, baccharin and DCBEN were detected.

Considering the interaction between *B. dracunculifolia* and *A. mellifera* in the production of green propolis, it is interesting to note that, according to Lima [34], the optimum time of the year for the highest yield of BGP production was from December to April. Sousa et al. [23] demonstrate that the yield of essential oil from *B. dracunculifolia* leaves is higher from February to April. It is important to point out that the relationship between period of leafbud growing and contents of physiological compounds was confirmed by this experiment, once the period of leafbud growth was coincident with the period of higher content of secondary metabolites, which corresponds to rainfall season. Therefore, the optimum time for both green propolis and *B. dracunculifolia* essential oil production was the same for phenolic compounds production as well. Hence, it is suggested that the best time to obtain good qualitative and quantitative results, considering phenolic compounds, essential oil and green propolis production is mainly between December and April, which matches with the summer time. All populations were cultivated, and the population from Colombo 1 produced the highest yield of dry biomass and good concentration of AME, but it produced the lowest amounts of other important compounds, such as artepillin C and isosakuranetin.

The cultivation of *B. dracunculifolia* is economically viable, and it can be scaled up for commercial production, since the biomass production, mean yields of crude extract and essential oils, as well as phenolic compounds were excellent.

## Funding 

FAPESP (Grants, No. 04/13005-1; 06/59893-0; 01/14219-7); and CAPES (Grant. PDEE/BEX 0387/04-5), Brazil.

## Figures and Tables

**Table 1:** Collection sites of the fruits of *B. dracunculifolia* and their coordinates.

State	Regions	Latitude	Longitude	Altitude
		(S)	(W)	(m)
São Paulo	Franca 1	20^˚^ 32′ 19′′	47^˚^ 24′ 03′′	996
	Cajurú	21^˚^ 16′ 31′′	47^˚^ 18′ 15′′	775
	Franca 2	20^˚^ 15′ 25′′	47^˚^ 28′ 36′′	1035
	Ribeirão Preto	21^˚^ 10′ 39′′	47^˚^ 48′ 37′′	546
	Campinas	22^˚^ 54′ 20′′	47^˚^ 03′ 39′′	854

Minas Gerais	Alfenas	21^˚^ 25′ 45′′	45^˚^ 56′ 50′′	881
	Paraguaçu	21^˚^ 32′ 50′′	45^˚^ 44′ 15′′	826
	Ouro Fino	22^˚^ 16′ 59′′	46^˚^ 22′ 08′′	908

Paraná	Colombo 1	25^˚^ 17′ 30′′	49^˚^ 13′ 27′′	1027
	Colombo 2	25^˚^ 19′ 29′′	49^˚^ 18′ 36′′	945

**Table 2:** Effect of seasonality on caffeic acid content (%) of leaves of *B. dracunculifolia* along 1 year.

Month/	São Paulo state					Minas Gerais state			Paraná state	
regions	Franca 1	Cajurú	Franca 2	Ribeirão Preto	Campinas	Paraguaçu	Alfenas	Ouro Fino	Colombo 1	Colombo 2
May/04^(A)^	3.70 ± 0.20^(a)^	3.41 ± 0.21^(a)^	3.57 ± 0.25^(a)^	*3.50* ± *0.28* ^(a)^	3.15 ± 0.08^(a)^	3.45 ± 0.18^(a)^	3.70 ± 0.23^(a)^	3.45 ± 0.14^(a)^	4.09 ± 0.41^(a)^	3.51 ± 0.37^(a)^
June/04^(A)^	4.32 ± 0.18^(a)^	4.14 ± 0.13^(a)^	3.96 ± 0.24^(a)^	*3.95* ± *0.21* ^(a)^	4.29 ± 0.11^(a)^	4.68 ± 0.49^(b)^	3.99 ± 0.11^(a)^	4.07 ± 0.38^(b)^	3.99 ± 0.11^(a)^	4.14 ± 0.10^(b)^
July/04^(A)^	5.07 ± 0.56^(b)^	4.09 ± 0.69^(a)^	4.42 ± 0.30^(a)^	3.58 ± 0.77^(a)^	4.01 ± 0.42^(a)^	4.41 ± 0.15^(a)^	4.66 ± 0.80^(b)^	3.53 ± 0.59^(a)^	4.90 ± 0.16^(b)^	4.67 ± 0.15^(b)^
August/04^(A)^	4.37 ± 0.38^(a)^	3.40 ± 0.65^(a)^	4.10 ± 0.11^(a)^	4.09 ± 0.14^(a)^	4.24 ± 0.40^(a)^	3.74 ± 0.35^(a)^	4.24 ± 0.49^(a)^	3.54 ± 0.36^(a)^	4.57 ± 0.87^(a)^	3.34 ± 0.78^(a)^
Sept/04^(A)^	3.40 ± 0.35^(a)^	3.12 ± 0.20^(a)^	3.25 ± 0.68^(a)^	3.03 ± 0.48^(a)^	3.99 ± 0.15^(a)^	3.58 ± 0.45^(a)^	2.94 ± 0.16^(a)^	2.71 ± 0.45^(a)^	3.81 ± 0.91^(a)^	2.53 ± 0.81^(a)^
Oct/04^(B)^	4.29 ± 0.76^(a)^	4.35 ± 0.10^(b)^	5.57 ± 0.83^(b)^	4.82 ± 0.88^(b)^	6.00 ± 0.62^(b)^	5.68 ± 1.11^(b)^	4.11 ± 0.31^(a)^	4.55 ± 0.81^(b)^	6.50 ± 1.97^(b)^	3.71 ± 1.70^(a)^
Nov/04^(A)^	4.18 ± 0.34^(a)^	4.05 ± 0.09^(a)^	4.49 ± 0.47^(b)^	3.58 ± 0.36^(a)^	4.24 ± 0.46^(a)^	4.41 ± 0.28^(a)^	4.02 ± 0.21^(a)^	4.32 ± 0.20^(b)^	5.14 ± 1.28^(b)^	3.33 ± 1.15^(a)^
Dec/04^(A)^	3.86 ± 0.18^(a)^	3.47 ± 0.28^(a)^	3.44 ± 0.13^(a)^	3.41 ± 0.23^(a)^	3.6 ± 0.55^(a)^	4.02 ± 0.04^(a)^	3.96 ± 0.20^(a)^	3.73 ± 0.16^(a)^	3.70 ± 0.25^(a)^	3.34 ± 0.23^(b)^
Jan/05^(B)^	5.35 ± 0.46^(b)^	4.70 ± 0.46^(b)^	5.24 ± 0.72^(b)^	4.85 ± 0.51^(b)^	5.87 ± 0.28^(b)^	4.31 ± 0.61^(a)^	5.17 ± 0.17^(b)^	5.00 ± 0.46^(b)^	4.76 ± 0.52^(b)^	4.02 ± 0.47^(b)^
Feb/05^(A)^	4.65 ± 0.90^(b)^	3.76 ± 0.63^(a)^	3.32 ± 0.75^(a)^	4.60 ± 0.60^(b)^	5.66 ± 0.65^(b)^	4.57 ± 1.00^(a)^	5.30 ± 0.59^(b)^	4.47 ± 1.50^(b)^	3.42 ± 1.00^(a)^	4.06 ± 0.90^(b)^
March/05^(B)^	*5.58* ± *0.33* ^(b)^	5.06 ± 0.37^(b)^	5.58 ± 0.51^(b)^	5.09 ± 0.37^(b)^	5.81 ± 0.29^(b)^	4.87 ± 0.53^(b)^	5.62 ± 0.42^(b)^	5.03 ± 0.40^(b)^	3.97 ± 0.77^(a)^	5.06 ± 0.70^(b)^
April/05^(B)^	*4.23* ± *0.47* ^(a)^	4.34 ± 0.08^(b)^	4.36 ± 0.17^(b)^	5.03 ± 0.54^(b)^	5.27 ± 0.39^(b)^	4.07 ± 0.23^(a)^	4.39 ± 0.13^(b)^	4.20 ± 0.22^(b)^	3.67 ± 0.10^(a)^	3.78 ± 0.11^(b)^

Capital and small letters denote significant differences at *P* < .01. (A) and (B) refer to the mean values among population for each month; (a) and (b) correspond to the significant differences for each population in time frame studied.

**Table 3:** Effect of seasonality on AME content (%) of leaves of *B. dracunculifolia* along 1 year.

Month/	São Paulo state					Minas Gerais state			Paraná state	
regions	Franca 1^(I)^	Cajurú^(I)^	Franca 2^(I)^	Ribeirão Preto^(I)^	Campinas^(I)^	Paraguaçu^(I)^	Alfenas^(I)^	Ouro Fino^(I)^	Colombo 1^(II)^	Colombo 2^(I)^
May/04^(B)^	0.64 ± 0.06^(b)^	0.73 ± 0.07^(b)^	0.78 ± 0.08^(b)^	0.60 ± 0.07^(b)^	0.72 ± 0.08^(b)^	0.49 ± 0.22^(b)^	0.80 ± 0.02^(b)^	0.83 ± 0.19^(b)^	0.79 ± 0.23^(b)^	0.47 ± 0.11^(b)^
June/04^(A)^	0.29 ± 0.01^(a)^	0.30 ± 0.06^(a)^	0.40 ± 0.05^(a)^	0.29 ± 0.05^(a)^	0.30 ± 0.01^(a)^	0.29 ± 0.07^(a)^	0.39 ± 0.12^(a)^	0.56 ± 0.14^(b)^	0.45 ± 0.13^(a)^	0.27 ± 0.06^(a)^
July/04^(A)^	0.32 ± 0.02^(a)^	0.29 ± 0.02^(a)^	0.29 ± 0.02^(a)^	0.26 ± 0.03^(a)^	0.25 ± 0.01^(a)^	0.20 ± 0.08^(a)^	0.31 ± 0.04^(a)^	0.32 ± 0.07^(a)^	0.56 ± 0.21^(a)^	0.27 ± 0.10^(a)^
August/04^(A)^	0.39 ± 0.01^(a)^	0.38 ± 0.02^(a)^	0.42 ± 0.03^(a)^	0.34 ± 0.09^(a)^	0.20 ± 0.10^(a)^	0.18 ± 0.14^(a)^	0.38 ± 0.01^(a)^	0.37 ± 0.11^(a)^	0.72 ± 0.16^(b)^	0.50 ± 0.08^(b)^
Sept/04^(B)^	0.91 ± 0.16^(b)^	0.69 ± 0.11^(b)^	0.79 ± 0.13^(b)^	0.62 ± 0.13^(b)^	0.59 ± 0.02^(b)^	0.37 ± 0.27^(b)^	0.76 ± 0.09^(b)^	0.89 ± 0.28^(b)^	1.11 ± 0.22^(b)^	0.80 ± 0.11^(b)^
Oct/04^(B)^	0.51 ± 0.16^(b)^	0.29 ± 0.16^(a)^	0.59 ± 0.13^(b)^	0.43 ± 0.11^(b)^	0.45 ± 0.01^(b)^	0.43 ± 0.02^(b)^	0.46 ± 0.01^(b)^	0.45 ± 0.02^(b)^	0.92 ± 0.21^(b)^	0.63 ± 0.10^(b)^
Nov/04^(A)^	0.38 ± 0.04^(a)^	0.32 ± 0.03^(a)^	0.32 ± 0.05^(a)^	0.27 ± 0.04^(a)^	0.32 ± 0.04^(a)^	0.24 ± 0.04^(a)^	0.30 ± 0.04^(a)^	0.25 ± 0.03^(a)^	0.90 ± 0.29^(b)^	0.49 ± 0.14^(b)^
Dec/04^(A)^	0.22 ± 0.04^(a)^	0.27 ± 0.06^(a)^	0.33 ± 0.06^(a)^	0.20 ± 0.08^(a)^	0.39 ± 0.13^(a)^	0.23 ± 0.05^(a)^	0.24 ± 0.02^(a)^	0.21 ± 0.02^(a)^	0.88 ± 0.23^(b)^	0.56 ± 0.11^(b)^
Jan/05^(A)^	0.20 ± 0.11^(a)^	0.35 ± 0.13^(a)^	0.45 ± 0.11^(a)^	0.29 ± 0.09^(a)^	0.32 ± 0.02^(a)^	0.13 ± 0.01^(a)^	0.15 ± 0.01^(a)^	0.14 ± 0.01^(a)^	0.92 ± 0.34^(b)^	0.44 ± 0.17^(b)^
Feb/05^(B)^	0.81 ± 0.12^(b)^	0.64 ± 0.10^(b)^	0.81 ± 0.15^(b)^	0.49 ± 0.13^(b)^	0.67 ± 0.13^(b)^	0.81 ± 0.03^(b)^	0.85 ± 0.13^(b)^	0.67 ± 0.09^(b)^	0.91 ± 0.30^(b)^	0.06 ± 0.30^(a)^
March/05^(B)^	0.61 ± 0.05^(b)^	0.54 ± 0.06^(b)^	0.66 ± 0.06^(b)^	0.55 ± 0.05^(b)^	0.56 ± 0.01^(b)^	0.66 ± 0.06^(b)^	0.58 ± 0.09^(b)^	0.71 ± 0.07^(b)^	0.86 ± 0.35^(b)^	0.37 ± 0.17^(b)^
April/05^(B)^	0.67 ± 0.16^(b)^	0.45 ± 0.17^(b)^	0.79 ± 0.14^(b)^	0.57 ± 0.13^(b)^	0.56 ± 0.03^(b)^	0.72 ± 0.02^(b)^	0.75 ± 0.06^(b)^	0.67 ± 0.04^(b)^	0.90 ± 0.29^(b)^	0.47 ± 0.15^(b)^

Capital and small letters denote significant differences at *P* < .01. (A) and (B) refer to the mean values among population for each month; (a) and (b) correspond to the significant differences for each population in time frame studied. (I) and (II) denote significant differences at *P* < .05 among populations in time frame studied.

**Table 4:** Effect of seasonality on isosakuranetin content (%) of leaves of *B. dracunculifolia* along 1 year.

Month/	São Paulo state					Minas Gerais state			Paraná state	
regions	Franca 1^(I)^	Cajurú^(I)^	Franca 2^(I)^	Ribeirão Preto^(I)^	Campinas^(I)^	Paraguaçu^(II)^	Alfenas^(I)^	Ouro Fino^(I)^	Colombo 1^(II)^	Colombo 2^(I)^
May/04^(A)^	0.00^(a)^	0.00^(a)^	0.00^(a)^	0.00^(a)^	0.00^(a)^	0.00^(a)^	0.00^(a)^	0.00^(a)^	0.00^(a)^	0.00^(a)^
June/04^(A)^	0.00^(a)^	0.00^(a)^	0.00^(a)^	0.00^(a)^	0.00^(a)^	0.00^(a)^	0.00^(a)^	0.00^(a)^	0.00^(a)^	0.00^(a)^
July/04^(A)^	0.00^(a)^	0.00^(a)^	0.00^(a)^	0.00^(a)^	0.00^(a)^	0.00^(a)^	0.00^(a)^	0.00^(a)^	0.00^(a)^	0.00^(a)^
August/04^(A)^	0.49 ± 0.17^(b)^	0.00^(a)^	0.00^(a)^	0.37 ± 0.05^(b)^	0.00^(a)^	0.71 ± 0.16^(b)^	0.34 ± 0.01^(b)^	0.35 ± 0.11^(b)^	0.00^(a)^	0.00^(a)^
Sept/04^(A)^	0.53 ± 0.20^(b)^	0.00^(a)^	0.37 ± 0.08^(b)^	0.50 ± 0.06^(b)^	0.45 ± 0.06^(b)^	1.01 ± 0.23^(b)^	0.69 ± 0.05^(b)^	0.62 ± 0.21^(b)^	0.00^(a)^	0.00^(a)^
Oct/04^(A)^	0.00^(a)^	0.00^(a)^	0.34 ± 0.24^(b)^	0.48 ± 0.03^(b)^	0.40 ± 0.04^(b)^	0.86 ± 0.17^(b)^	0.48 ± 0.02^(b)^	0.45 ± 0.23^(b)^	0.00^(a)^	0.26 ± 0.03^(b)^
Nov/04^(B)^	0.61 ± 0.08^(b)^	0.50 ± 0.05^(b)^	0.56 ± 0.24^(b)^	0.56 ± 0.01^(b)^	0.57 ± 0.02^(b)^	1.19 ± 0.26^(b)^	0.68 ± 0.02^(b)^	0.65 ± 0.30^(b)^	0.00^(a)^	0.26 ± 0.02^(b)^
Dec/04^(B)^	0.92 ± 0.26^(b)^	0.55 ± 0.04^(b)^	0.49 ± 0.05^(b)^	0.59 ± 0.04^(b)^	0.61 ± 0.01^(b)^	1.18 ± 0.28^(b)^	0.79 ± 0.10^(b)^	0.65 ± 0.27^(b)^	0.00^(a)^	0.25 ± 0.10^(b)^
Jan/05^(B)^	0.74 ± 0.16^(b)^	0.52 ± 0.01^(b)^	0.50 ± 0.19^(b)^	0.47 ± 0.07^(b)^	0.53 ± 0.04^(b)^	0.78 ± 0.16^(b)^	0.55 ± 0.02^(b)^	0.58 ± 0.13^(b)^	0.00^(a)^	0.26 ± 0.02^(b)^
Feb/05^(B)^	1.11 ± 0.25^(b)^	0.75 ± 0.04^(b)^	0.81 ± 0.12^(b)^	0.81 ± 0.02^(b)^	0.86 ± 0.12^(b)^	1.27 ± 0.08^(b)^	1.16 ± 0.27^(b)^	0.78 ± 0.26^(b)^	0.00^(a)^	0.35 ± 0.05^(b)^
March/05^(B)^	0.89 ± 0.06^(b)^	0.80 ± 0.08^(b)^	0.69 ± 0.16^(b)^	0.86 ± 0.26^(b)^	0.83 ± 0.10^(b)^	1.32 ± 0.32^(b)^	0.87 ± 0.07^(b)^	0.77 ± 0.29^(b)^	0.00^(a)^	0.36 ± 0.07^(b)^
April/05^(B)^	0.16 ± 0.09^(a)^	0.67 ± 0.05^(b)^	0.60 ± 0.20^(b)^	0.75 ± 0.03^(b)^	0.79 ± 0.05^(b)^	1.40 ± 0.28^(b)^	0.86 ± 0.08^(b)^	0.74 ± 0.25^(b)^	0.00^(a)^	0.47 ± 0.08^(b)^

Capital and small letters denote significant differences at *P* < .01. (A) and (B) refer to the mean values among population for each month; (a) and (b) correspond to the significant differences for each population in time frame studied. (I) and (II) denote significant differences at *P* < .05 among populations in time frame studied.

**Table 5:** Effect of seasonality on artepillin C content (%) of leaves of *B. dracunculifolia* along 1 year.

Month/	São Paulo state					Minas Gerais state			Paraná state	
regions	Franca 1^(I)^	Cajurú^(I)^	Franca 2^(I)^	Ribeirão Preto^(I)^	Campinas^(I)^	Paraguaçu^(I)^	Alfenas^(I)^	Ouro Fino^(I)^	Colombo 1^(II)^	Colombo 2^(I)^
May/04^(a)^	0.04 ± 0.01^(a)^	0.04 ± 0.01^(a)^	0.03 ± 0.02^(a)^	0.00^(a)^	0.04 ± 0.02^(a)^	0.04 ± 0.01^(a)^	0.03 ± 0.01^(a)^	0.04 ± 0.01^(a)^	0.00^(a)^	0.00^(a)^
June/04^(a)^	0.11 ± 0.02^(a)^	0.08 ± 0.06^(a)^	0.15 ± 0.03^(b)^	0.08 ± 0.03^(a)^	0.10 ± 0.01^(a)^	0.11 ± 0.02^(a)^	0.14 ± 0.02^(a)^	0.10 ± 0.03^(a)^	0.00^(a)^	0.03 ± 0.01^(a)^
July/04^(a)^	0.15 ± 0.07^(b)^	0.05 ± 0.01^(a)^	0.15 ± 0.05^(b)^	0.10 ± 0.05^(a)^	0.16 ± 0.04^(b)^	0.12 ± 0.01^(a)^	0.11 ± 0.01^(a)^	0.10 ± 0.01^(a)^	0.00^(a)^	0.06 ± 0.02^(a)^
August/04^(a)^	0.10 ± 0.01^(a)^	0.09 ± 0.02^(a)^	0.10 ± 0.01^(a)^	0.11 ± 0.02^(a)^	0.13 ± 0.01^(a)^	0.15 ± 0.04^(b)^	0.10 ± 0.03^(a)^	0.09 ± 0.01^(a)^	0.00^(a)^	0.03 ± 0.01^(a)^
Sept/04^(a)^	0.10 ± 0.01^(a)^	0.11 ± 0.07^(a)^	0.13 ± 0.02^(a)^	0.09 ± 0.02^(a)^	0.14 ± 0.04^(a)^	0.16 ± 0.01^(b)^	0.17 ± 0.02^(b)^	0.19 ± 0.02^(b)^	0.00^(a)^	0.04 ± 0.01^(a)^
Oct/04^(a)^	0.13 ± 0.04^(a)^	0.19 ± 0.10^(b)^	0.26 ± 0.05^(b)^	0.21 ± 0.05^(b)^	0.20 ± 0.01^(b)^	0.28 ± 0.04^(b)^	0.23 ± 0.03^(b)^	0.22 ± 0.03^(b)^	0.00^(a)^	0.13 ± 0.05^(b)^
Nov/04^(b)^	0.26 ± 0.04^(b)^	0.31 ± 0.05^(b)^	0.45 ± 0.11^(b)^	0.20 ± 0.09^(b)^	0.31 ± 0.08^(b)^	0.35 ± 0.04^(b)^	0.29 ± 0.03^(b)^	0.30 ± 0.01^(b)^	0.06 ± 0.01^(b)^	0.10 ± 0.02^(b)^
Dec/04^(b)^	0.41 ± 0.06^(b)^	0.32 ± 0.02^(b)^	0.38 ± 0.08^(b)^	0.23 ± 0.09^(b)^	0.22 ± 0.01^(b)^	0.36 ± 0.01^(b)^	0.37 ± 0.06^(b)^	0.26 ± 0.08^(b)^	0.05 ± 0.02^(b)^	0.15 ± 0.04^(b)^
Jan/05^(b)^	0.46 ± 0.02^(b)^	0.49 ± 0.12^(b)^	0.50 ± 0.15^(b)^	0.18 ± 0.14^(b)^	0.29 ± 0.08^(b)^	0.36 ± 0.01^(b)^	0.38 ± 0.04^(b)^	0.31 ± 0.05^(b)^	0.05 ± 0.02^(b)^	0.18 ± 0.05^(b)^
Feb/05^(b)^	0.53 ± 0.04^(b)^	0.47 ± 0.05^(b)^	0.71 ± 0.12^(b)^	0.44 ± 0.11^(b)^	0.58 ± 0.10^(b)^	0.50 ± 0.15^(b)^	0.71 ± 0.13^(b)^	0.46 ± 0.18^(b)^	0.08 ± 0.03^(b)^	0.26 ± 0.06^(b)^
March/05^(b)^	0.46 ± 0.01^(b)^	0.47 ± 0.09^(b)^	0.55 ± 0.04^(b)^	0.51 ± 0.05^(b)^	0.59 ± 0.06^(b)^	0.53 ± 0.01^(b)^	0.55 ± 0.05^(b)^	0.45 ± 0.07^(b)^	0.10 ± 0.05^(b)^	0.37 ± 0.10^(b)^
April/05^(b)^	0.72 ± 0.01^(b)^	0.71 ± 0.07^(b)^	0.87 ± 0.08^(b)^	0.73 ± 0.16^(b)^	1.09 ± 0.25^(b)^	0.77 ± 0.13^(b)^	0.96 ± 0.16^(b)^	0.65 ± 0.22^(b)^	0.16 ± 0.05^(b)^	0.45 ± 0.10^(b)^

Capital and small letters denote significant differences at *P* < .01. (A) and (B) refer to the mean values among population for each month; (a) and (b) correspond to the significant differences for each population in time frame studied. (I) and (II) denote significant differences at *P* < .05 among populations in time frame studied.
